# Radial-Probe Endobronchial Ultrasound as Part of Different Navigational Bronchoscopy Modalities in Combination with Cryobiopsy Could Be More than a Confirmation Tool: A Case Series

**DOI:** 10.3390/diagnostics15151884

**Published:** 2025-07-27

**Authors:** Nevenka Piskac Zivkovic, Maja Karaman Ilic, Suncana Divosevic, Hrvoje Feljan, Igor Nikolic, Zrinka Juros, Ana-Marija Sola, Sven Seiwerth, Dragan Schwarz, Ivica Mazuranic

**Affiliations:** 1 Special Hospital Radiochirurgia Zagreb, 10431 Sveta Nedelja, Croatia; maja.karaman.ilic@radiochirurgia.hr (M.K.I.); suncana.divosevic@radiochirurgia.hr (S.D.); hrvoje.feljan@gmail.com (H.F.); inik21257@gmail.com (I.N.); dragan.schwarz@radiochirurgia.hr (D.S.); ivicamazuranic@gmail.com (I.M.); 2 Faculty of Pharmacy and Biochemistry, University of Zagreb, 10000 Zagreb, Croatia; 3 Faculty of Dental Medicine and Health, University of Osijek, 31000 Osijek, Croatia; 4 Faculty of Medicine Osijek, University of Osijek, 31000 Osijek, Croatia; 5 Sestre Milosrdnice University Hospital Center, 10000 Zagreb, Croatia; zrinka.juros@gmail.com; 6 Special Hospital for Lung Diseases, 10000 Zagreb, Croatia; anamarija.hr@gmail.com; 7 School of Medicine, University of Zagreb, 10000 Zagreb, Croatia; sven.seiwerth@mef.hr

**Keywords:** PPNs, lung cancer, cryobiopsy, rEBUS, eccentric lesion

## Abstract

**Background**: As part of different navigational bronchoscopy (NVB) modalities, radial-probe endobronchial ultrasound (rEBUS) is used to confirm the peribronchial localization of peripheral pulmonary nodules (PPNs) immediately before collecting samples for histopathological analysis. **Methods**: This retrospective case series study presents the results of en bloc cryobiopsy of PPNs using a flexible 1.1-mm cryoprobe with different NVB modalities. For PPNs classified as adjacent or eccentric lesions by rEBUS (ES-rEBUS), the cryoprobe’s position was adjusted by 90–180° in relation to the ultrasound image of the lesion during the first and second biopsies. **Results**: All patients with a final histopathologically confirmed diagnosis of PPNs had positive rEBUS findings, regardless of the navigation modality, eccentric (18/42 patients, 43%) and concentric (24/42 patients, 57%) rEBUS view. In 5 out of 6 patients without a histopathological diagnosis, PPNs were not visualized by radial ultrasound. In the (ES-rEBUS) group of patients, 4 out of 18 had fewer than three biopsy samples collected per procedure, which means only an adjusted probe position has been applied, although diagnostic outcomes were achieved. Common Terminology Criteria for Adverse Events (CTCAE) grade 2 complications were reported in 10.4% of the patients, and grade 3 complications in 2% of the patients. **Conclusions**: Confirming the localization of nodules by rEBUS and properly adjusting the cryoprobe immediately before cryobiopsy of PPNs resulted in a diagnostic yield meeting the literature standards.

## 1. Introduction

Early-stage lung tumors found on low-dose thoracic CT (LDCT) are peripheral pulmonary nodules (PPNs) with a diameter of up to 40 mm, surrounded by healthy lung parenchyma [1]. The prevalence of PPNs in lung cancer screening programs ranges from 17 to 54% [2]. Nodules of moderateto-high risk for malignant disease (1.5–3.5%) detected in lung cancer screening programs are indicated for further diagnostic workup and histopathological analysis [3,4]. Endobronchial access is the first method of choice rather than transthoracic biopsy due to fewer complications, which includes the possibility of mediastinal lymph node biopsy being performed during the same procedure (concomitant EBUS) [5]. If during flexible bronchoscopy additional diagnostic tools are used (image-guided bronchoscopy, IGB), diagnostic success rates increase from 49% (conventional bronchoscopy) to 83% (navigational bronchoscopy) [6,7,8,9,10,11]. According to meta-analyses, rEBUS has an up to 70% diagnostic yield for PPNs [12]. As part of different modalities of image-guided bronchoscopy, rEBUS is used to confirm the peribronchial localization of nodules immediately before tissue collection, even with advanced navigational bronchoscopy modalities such as electromagnetic navigation and robotic bronchoscopy [10,11,13,14,15]. Using rEBUS before cryobiopsy (CB) with a 1.1-mm flexible cryoprobe, the diagnostic yield of NVB is additionally improved [16,17,18,19,20,21], in particular for the more difficult nodule group (<20 mm, ground glass opacity (GGO) nodules, nodules classified as eccentric or adjacent to lesions by rEBUS). The formal definitions of concentric, eccentric, and adjacent rEBUS views in the context of cryobiopsy refer to the orientation of the cryoprobe relative to the target lesion as visualized by radial endobronchial ultrasound (rEBUS). A “concentric” orientation means the probe is within and surrounded by the lesion. An “eccentric” orientation indicates the probe is within the lesion but biased toward one side. “Adjacent” means the probe is next to the lesion but not within it. 

This paper presents the results of cryobiopsy of PPNs as part of different NVB modalities. For PPNs classified as adjacent or eccentric lesions by radial endobronchial ultrasound (ES-rEBUS), the position of the cryoprobe was adjusted by 90–180° in relation to the ultrasound image of the lesion during the first and second biopsies. This retrospective case series study aimed at determining whether changes in the tissue collection method affect (a) the overall diagnostic yield of the method, and (b) the incidence of complications according to the CTCAE criteria.

## 2. Materials and Methods

### 2.1. Study Design

This retrospective case series study was performed for the period of one year (December 2023–December 2024). This study included patients aged 18 and above who underwent cryobiopsy of PPNs using a flexible 1.1-mm cryoprobe in various NVB modalities. Patients with PPNs greater than 40 mm were excluded from this study. For ES-rEBUS verified lesions, the position of the flexible cryoprobe was adjusted by 90–180° in the direction of radial-probe rotation.

A retrospective analysis of patient data was approved by the Ethics Committee of the institution where this study was performed. All the procedures were carried out according to the 2000 Declaration of Helsinki, and in line with the 2002 and 2004 amendments. Missing data, such as the results of the surgical procedure, were subsequently obtained during a control visit in the hospital.

### 2.2. The rEBUS Diagnostic Method

Prior to a decision by the multidisciplinary team (MDT), all patients underwent MSCT and PET/CT scans of the thorax. Preparation for the diagnostic procedure as well as the procedure itself were accomplished after obtaining the patient’s informed consent.

All the procedures were performed using a Univent 8.0–8.5 endotracheal tube with an endobronchial blocker, under total intravenous anesthesia (TIVA). Patients classified as ASA 4 by the American Society of Anesthesiologists were excluded from this study. In the case of electromagnetic navigation (DT-ENB), during peripheral airway visualization, the positive end-expiratory pressure (PEEP) was increased to 8–10 cm H_2_O by recruitment maneuver prior to the procedure.

The procedures were performed using a flexible 1.1-mm cryoprobe (ERBECRYO^TM^ 2) on the cryobiopsy device ERBECRYO^TM^ 2 (ERBE, Medizintechnik, Tübingen, Germany).

Once the peribronchial location of the nodule is confirmed using rEBUS, the ultrasound probe is removed from the working channel, and the cryoprobe is introduced. The tip of the cryoprobe is placed against the bronchial wall before activating the “freezing” time. The target tissue can be frozen to the cryoprobe either directly (en face), at an angle (tangentially), or completely around (circumferentially, 360°). This allows for flexibility in positioning the cryoprobe at either 90° or 180° in relation to the ultrasound image of eccentric or adjacent peribronchial lesions. Based on published recommendations [16,17,18,19,20,21] and our experience with diffuse parenchymal lung disease, freezing time was 5–7 s, followed by en bloc biopsy and preventive endobronchial blocker inflation.

The concentric, adjacent, or eccentric rEBUS views of peribronchial lesions are classified as a rEBUS-positive sign.

**Figure 1:**
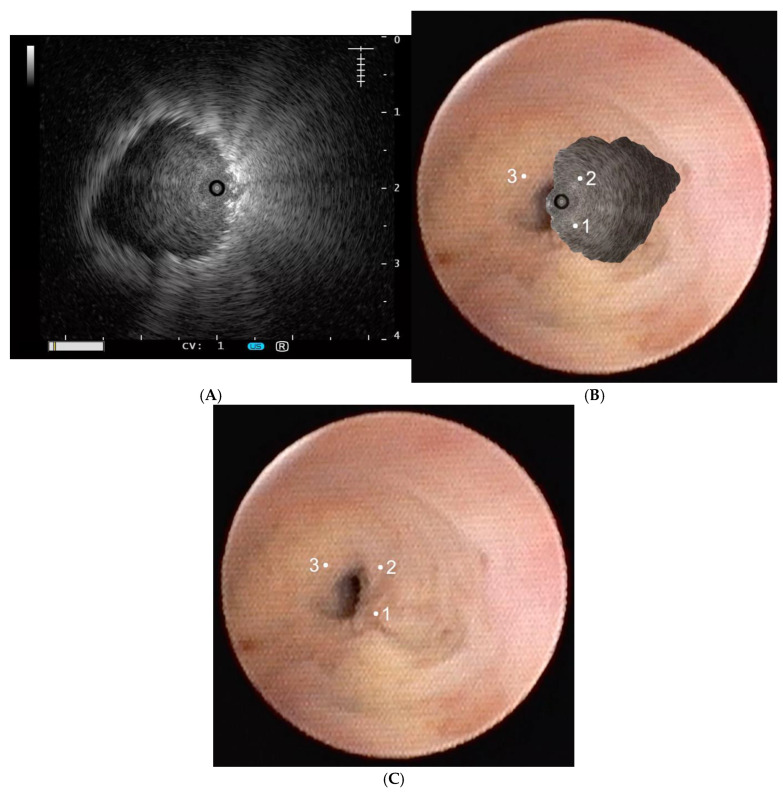
(**A**) Lung nodule classified as eccentric lesion by rEBUS. (**B**,**C**) A detailed procedure description.

In the cases of adjacent or eccentric rEBUS views of peribronchial lesions (ES-rEBUS), the position of the cryoprobe was adjusted as follows: (A) first biopsy—180° in relation to the ultrasound image of the lesion; (B) second biopsy—90° in relation to the ultrasound image of the lesion; and (C) third biopsy—on the location of the ultrasound image of the peribronchial lesion (Figure 1). Imprint cytology was performed on all the specimens. All cryobiopsy samples were analyzed using fast cytology during the procedure. Rapid On-site Evaluation (ROSE) diagnostic results indicate that the pathologist can classify the sample as either benign, malignant, or potentially malignant. The five additional needle samples were taken with a peripheral PeriView Flex needle, FLEX-TBNA (21 G, Olympus, Tokyo, Japan, NA-403D) according to the interventional pulmonologist’s decision, primarily in cases of increased bleeding after the first biopsy or non-diagnostic ROSE results. In those cases, the needle puncture position was adjusted with the position of the cryoprobe.

Patients in whom PPNs were not histopathologically verified by any of the mentioned diagnostic methods, and who still had a moderate or high probability of malignant disease, were referred for surgical intraoperative biopsy and further treatment depending on the results (segmentectomy, lobectomy).

Once the peribronchial location of the nodule is confirmed, the ultrasound probe is removed from the working channel, and the cryoprobe is introduced. The tip or lateral surface of the cryoprobe is placed against the bronchial wall before activating the “freezing” time. After freezing for either 5 or 7 s, the en bloc biopsy is performed. The objective is to obtain at least three cryobiopsies from each patient. For patients with lung nodules classified as adjacent or eccentric rEBUS signs (ES-rEBUS), the positioning of the cryoprobe is adjusted within the image of the rEBUS as follows: (1) first biopsy—180° in relation to the ultrasound image of the lesion (obtained by rEBUS); (2) second biopsy—90° in relation to the ultrasound image of the lesion; (3) third biopsy—on the location of the ultrasound image of the peribronchial lesion.

### 2.3. Choice of Image-Guided Bronchoscopy (IGB) Navigational Methods According to Pulmonary Nodule Localization

For PPNs of the intermediate zone of lung parenchyma:

1. Virtual bronchoscopic navigation (VBN), Siemens Healthcare, Forchheim, Germany;
2. Ultrathin bronchoscope (UTB), Olympus Medical BF-MP190F, Tokyo, Japan, OD 3.0 mm, ID 1.7 mm;
3. Radial-probe endobronchial ultrasound (rEBUS), Olympus Medical UM-S20-17S, Tokyo, Japan.

**Figure 2:**
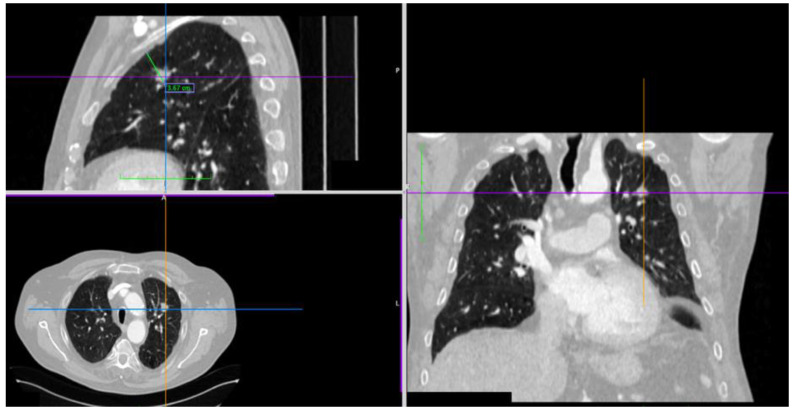
A solitary lung nodule on 2D and 3D MSCT chest reconstructions. Legend: The size of the nodule and distance from the visceral pleura to the inner edge of the nodule is 3.67 cm.

The intermediate zone is defined on 2D and 3D thoracic MSCT reconstructions as a distance of ≥3.5 cm from the visceral pleura to the inner edge of the nodule (endobronchially, 4th–7th bronchi branching subsegments) (Figure 2), and in cases of marked emphysema ≥ 4.0 cm (Cryo-Radial group, CRg).

For PPNs of the outer third of lung parenchyma:

1. DT-ENB (ILLUMISITE™ Platform, Minneapolis, MN, USA), Electromagnetic navigation (EMN) with integrated digital tomosynthesis (DT) for CT-to-body divergence correction. Our Center’s fluoroscopy device C-Arm (Ziehm Vision RFD 3D SN, Nuremberg, Germany) is used without a digital C-arm compatibility function to the Illumisite platform.
2. rEBUS, Olympus Medical (UM-S20-17S).

According to the MDT’s decision, the procedure is indicated for PPNs in the outer third of lung parenchyma (in 2D and 3D reconstructions) with a <3.5 cm distance from the inner edge of the nodule to the visceral pleura, and for which transthoracic puncture (TTP) is not indicated for the following reasons: nodule size < 10 mm; marked pulmonary emphysema; or inconvenient location in relation to the thoracic wall structures, primarily blood vessels (Illumisite-Cryo group, ILCg).

All patients were observed post-procedure at the clinic for an average of 2–4 h. A follow-up low-dose CT (LDCT) scan of the thorax was performed for the ILCg 2 h post-procedure, and for the CRg in case of a clinical indication (moderate post-procedural bleeding, inadequate recovery 2–4 h post-procedure).

CTCAE bleeding intensity classification: grade 1 (mild hemorrhage)—mild bleeding that requires suction, with or without local administration of adrenaline; grade 2 (moderate hemorrhage)—moderate symptoms, prolonged inflation of the blocker balloon (>1 min); grade 3 (severe hemorrhage)—significant bleeding requiring hospitalization, transfusion, or surgical intervention; grades 4 and 5 denote vital threat to the patient and death [5,21,22,23]. According to the same criteria, post-procedural pneumothorax is classified into 5 grades; placement of a thoracic drain is classified as grade 3 and belongs to the severe pneumothorax group.

### 2.4. Statistical Methods

Categorical data are represented by absolute and relative frequencies. Continuous variable distribution normality was tested by the Shapiro–Wilk test. Data were described by arithmetic mean and range. The statistical tool for data analysis used was MedCalc^®^ Statistical Software version 22.023 (MedCalc Software Ltd., Ostend, Belgium; https://www.medcalc.org; 30 April 2025).

## 3. Results

This retrospective case series study analyzed data from 48 patients with 8–40 mm PPNs who underwent different modalities of navigational bronchoscopy and CB using a 1.1-mm flexible cryoprobe. The position of the cryoprobe was adjusted by 90–180° in relation to PPNs classified as ES-rEBUS (Figure 1). Table 1 shows the demographic characteristics of the patients.

A diagnostic yield of 92% (33/36 patients) for the intermediate zone of lung parenchyma (CRg) was achieved by a combination of IGB modalities (VBN, UTB, and rEBUS). In this group, the average nodule size was 27.6 mm, and 34% of the patients were diagnosed with PPNs classified as ES-rEBUS. In the outer third of lung parenchyma (ILCg) with the applied IGB modality combination (DT-ENB and rEBUS), the diagnostic yield was 75% (9/12 patients). Average nodule size in this group was 12.5 mm, and 50% of the patients were diagnosed with PPNs classified as ES-rEBUS. Other patient characteristics (for the CRg and ILCg), as well as results of the diagnostic procedures, are shown in Table 1 and Table 2.

All 42/48 (87.5%) patients (CRg and ILCg) with a final histopathologically confirmed diagnosis of PPNs had positive rEBUS findings immediately prior to tissue collection by cryoprobe for histopathological analysis, regardless of the navigational modality and eccentric (18/42 patients, 43%) or concentric (24/42 patients, 57%) lesions found on rEBUS. In 88% (CRg and ILCg, 37/42) of patients with confirmed histopathological diagnosis, the cryobiopsy specimens were sufficient for histopathological evaluation and molecular testing. In 5/42 (12%) of patients, additional specimens obtained by conventional methods (imprint cytology and TBNA) were used to reach the final diagnosis. Twelve out of forty-eight (25%) patients, four of them with PPNs classified as ES-rEBUS, had fewer than three biopsy samples per procedure. In two patients, CB was indicated for next-generation sequencing (NGS).

Three out of thirty-six patients (8%) from the CRg had a non-diagnostic finding. In two patients, the nodule was not visible due to bronchial deformities and obstructed access to the newly arisen peripheral nodule. The mentioned deformities were due to a past episode of tuberculosis (one patient) and status after left lower lobectomy with atypical resection of the anterior segment of the left upper lobe due to cancer (one patient). In the third patient, the nodule was visible (classified as a concentric rEBUS view) but an adequate diagnostic specimen was not obtained. Due to moderate bleeding after the first CB, the sampling was continued by needle biopsy (FLEX-TBN) but the obtained specimens were insufficient to confirm the diagnosis.

Three out of twelve patients (25%) from the ILCg—in whom the nodule was not confirmed either as an eccentric or concentric lesion by rEBUS immediately prior to diagnostic tissue collection—had a non-diagnostic finding in spite of adequate PPN (nodule size 8–10 mm) localization by DT-ENB navigation. The TBNA method confirmed the presence of atypical cells (one patient), whereas in two patients, the findings were inconclusive.

The results of surgical biopsy in patients with unconfirmed histopathology for PPNs from samples obtained by any of the above-mentioned methods were as follows: one hamartoma, three adenocarcinomas, one planocellular carcinoma, and one metastatic carcinoma of the prostate.

### Complications

Moderate bleeding (CTCAE grade 2) was reported in four patients. All patients with moderate bleeding were from the Cryo-Radial group (CRg). Two patients had a histopathological finding of granulomatous inflammation, and two had adenocarcinoma. All four patients had PPNs classified as concentric lesions by rEBUS. One patient from the Illumisite-Cryo group (ILCg) was observed due to partial pneumothorax with spontaneous reabsorption. Overall, 5/48 (10.4%) patients had moderate (CTCAE grade 2) complications. Post-discharge hemoptysis was not reported, and follow-up thoracic LDCT after 24–48 h showed reabsorption of intra-alveolar hemorrhage > 50%. Pneumonia was not reported. Post-discharge, all patients with moderate hemorrhage were put on antibiotic therapy (levofloxacin, three patients; ciprofloxacin, one patient). In one patient from the CRg (1/48, 2%), significant bleeding (CTCAE grade 3) was reported in bullae formed in the right upper lobe of the lung parenchyma due to emphysema, and upper lobectomy was indicated. Histopathology of the biopsy specimen confirmed a granulomatous inflammation with caseous necrosis (rEBUS before cryobiopsy revealed concentric echo sign), and anti-TB treatment was introduced. During a prolonged incubation period, Mycobacterium avium complex (MAC) was isolated, and treatment proceeded according to protocol. At the 6-month follow-up, the patient had fully recovered.

## 4. Discussion

Radial endobronchial ultrasound (rEBUS) and correct cryo-probe adjustment (presented for the first time in our study results) immediately before the cryobiopsy of peripheral pulmonary nodules (PPNs) with adjacent or eccentric peribronchial localization may improve the diagnostic yield of different navigational bronchoscopy modalities. The presented retrospective case series study analyzed data from 48 patients with 8–40-mm PPNs who underwent navigational bronchoscopy with multiple modalities.

All CRg and ILCg patients (42/48, 87.5%) with a final histopathologically confirmed diagnosis of PPNs had a positive rEBUS finding immediately before tissue sampling, regardless of the navigation modality and whether the lesions found on rEBUS were classified as eccentric (18/42 patients, 43%) or concentric (24/42 patients, 57%). In 5/6 patients without histopathological diagnosis, PPNs were not visualized by rEBUS.

Radial-probe EBUS has been used for diagnostic purposes for PPNs since 2002 [24], starting as an image-guided diagnostic tool in combination with fluoroscopy. This diagnostic method had a lower diagnostic yield for more difficult nodules such as GGO nodules, small-sized (<20 mm) nodules, and nodules classified as eccentric in the rEBUS view. In a study by Chen et al. [25], out of a total of 467 nodules, 446 (96%) were successfully identified using rEBUS. When the radial-probe position was within the target lesion, the diagnostic yield was 84%, compared with 48% when the probe was positioned adjacent to the lesion. According to the results of a meta-analysis, rEBUS for PPNs has an overall diagnostic yield of 70% [12].

Cryobiopsy using a 1.1-mm flexible cryoprobe represents a contribution to the diagnostic success rates of different IGB methods for PPNs, including advanced navigational bronchoscopy methods such as RAB and DT-ENB [19,20], and especially in the cases of lesions classified as eccentric by radial ultrasound, GGO nodules, and <15-mm nodules (more difficult nodules). According to the study by Kho et al. [21], for eccentrically and adjacently oriented lesions, cryobiopsy significantly increased the diagnostic yield in comparison with forceps biopsy, from 48.8% (20 out of 41) to 75.0% (18 out of 24) (*p* < 0.05). In our study group, histopathology following cryobiopsy confirmed the diagnosis in all patients with lesions classified as ES-rEBUS. Due to an ultrasound probe rotation by 360°, the exact location of the peribronchial lesion could not be reliably determined [24,25]. However, based on long-term experience, in the case of PPNs distal from the fourth-generation bronchi branching (without bronchiectasis), targeted biopsy of the peribronchial lesion guided by rEBUS is possible. It is the adjacent and eccentric rEBUS views that enable confirmation of the clinical observation.

In patients with lesions classified as ES-rEBUS biopsy, samples were obtained as follows: (1) first biopsy—180° in relation to the ultrasound image of the lesion; (2) second biopsy—90° in relation to the ultrasound image of the lesion; and (3) third biopsy—on the location of the ultrasound image of the peribronchial lesion (Figure 1). In 4/18 patients, only one or two sample collections were performed; this means that, in four patients with ES-rEBUS, biopsy samples were collected only from positions at 180 or 90 degrees relative to the ultrasound image of the peribronchial nodule, yet diagnostic outcomes were successfully achieved. We assume that without appropriate cryoprobe adjustment, this group of patients would have non-diagnostic results.

According to our results, moderate complications by CTCAE grade were reported in 5/48 patients (10.5%). All patients with moderate bleeding were from the CRg and had lesions classified as concentric by rEBUS. In one patient from the CRg, significant bleeding was reported in the bullae formed in the right upper lobe of the lung parenchyma due to emphysema, and upper lobectomy was indicated. Histopathology confirmed a granulomatous inflammation, and the Mycobacterium avium complex (MAC) was isolated. Bullous emphysema has been described as a risk factor for the development of post-procedural complications such as pneumothorax and pulmonary hemorrhage [26], mainly as a consequence of the lack of parenchymal compression on a hemorrhaging blood vessel but also due to undiagnosed pulmonary hypertension as a consequence of the underlying disease. The mentioned safety profile of the diagnostic method is in accordance with the meta-analysis results for PPN cryobiopsy, which reports CTCAE grade 3 complications in 1.8% of patients [17].

Limitations of this retrospective case series study are the small number of patients and the lack of a control group. Our study results must be confirmed with randomized prospective studies. Likewise, the PPNs with concentric echo signs must be included in prospective studies to avoid blood vessels and reduce potential complications, mainly post-procedural hemorrhage.

## 5. Conclusions

Confirming the localization of nodules by rEBUS and properly adjusting the cryoprobe immediately before cryobiopsy of PPNs resulted in a diagnostic yield meeting the literature standards.

## Figures and Tables

**Table 1:** Radiological and ultrasound characteristics of peripheral pulmonary nodules (PPNs).

Diagnostic Procedures (Group of Patients)	Cryo-Radial Group (CRg)	Illumisite-Cryo Group (ILCg)
Patients, *n*	36	12
Gender distribution (M), *n* (%)	19 (53%)	6 (50%)
Age, mean (±SD)	67.7 ± SD 7.08	65.5 ± SD 7.56
Diameter of PPNs on CT scan (mm), mean (± SD)	27.6 ± SD 6.94	12.5 ± SD 3.45
Lobar location—right or left upper lobe, *n* (%)	23 (64%)	7 (58%)
Other locations, *n* (%)	13 (36%)	5 (42%)
Character of lesions, *n* (%)		
Solid	27 (75%)	8 (67%)
Part-solid	7 (19%)	4 (33%)
Ground glass opacity	2 (6%)	-
Positive bronchus sign, *n* (%)	24 (67%)	5 (42%)
ES-rEBUS *, *n* (%)	12 (34%)	6 (50%)

Legend: CRg (Cryo-Radial group)—combination of image-guided bronchoscopy (IGB) modalities used for nodules in the intermediate zone of the lung parenchyma, including virtual bronchoscopic navigation (VBN), ultrathin bronchoscope (UTB), radial-probe endobronchial ultrasound (rEBUS). ILCg (Illumisite-Cryo group)—DT-ENB (electromagnetic navigational bronchoscopy with integrated digital tomosynthesis) ILLUMISITE^™^ Platform and rEBUS were used as IGB modalities for nodules of the outer third of the lung parenchyma. * ES-rEBUS: PPNs classified as adjacent or eccentric lesions by rEBUS.

**Table 2:** The results of cryobiopsy (CB) of peripheral pulmonary nodules (PPNs) by different navigational bronchoscopy methods.

Cryobiopsy Results (PPNs)	Cryo-Radial (CRg)	Illumisite-Cryo (ILCg)
Sample size (mm), (range)	3–7	2–5
Time to first biopsy, * (min)	12 ± 5	22 ± 7
Diagnostic yield, n (%)	33/36 (92%)	9/12 (75%)
Malignant, n (%)	29/33 (88%)	5/9 (56%)
Pulmonary adenocarcinoma, n	16	3
Squamous cell carcinoma, n	9	1
** Others: NHL, meta colon, meta prostate, n	4	1
Benign, n (%)	4/33 (12%)	4/9 (44%)
Granulomatous inflammation, n	2	3
Hamartoma, n	1	0
Benign lymphocytic or neutrophilic infiltration, n	1	1
Complications, n (%)	5/36 (14 %)	1/12 (8%)
CTCAE grade 2, n	4	1
CTCAE grade 3, n	1	

Legend: CRg (Cryo-Radial group)—combination of image-guided bronchoscopy (IGB) modalities used for nodules in the intermediate zone of the lung parenchyma, including virtual bronchoscopic navigation (VBN), ultrathin bronchoscope (UTB), radial-probe endobronchial ultrasound (rEBUS). ILCg (Illumisite-Cryo group)—DT-ENB (electromagnetic navigational bronchoscopy with integrated digital tomosynthesis) ILLUMISITE™ Platform and rEBUS were used as IGB modalities for nodules of the outer third of the lung parenchyma. * Time to first biopsy indicates the time from bronchoscope insertion to obtaining the first biopsy sample. ** Other malignancies: NHL = 1, meta colon = 3, meta prostate = 1. Common Terminology Criteria for Adverse Events (CTCAE).

## Data Availability

The data presented in this study are available on request from the corresponding author. The data are not publicly available due to the hospital’s internal policies.

## The List of Abbreviations

Navigational bronchoscopy (NVB); Radial-probe endobronchial ultrasound (rEBUS); Peripheral pulmonary nodules (PPNs); PPNs classified as eccentric lesions by rEBUS (ES-rEBUS); Diagnostic yield (DY); Low-dose thoracic CT (LDCT); Ground glass opacity (GGO); Multidisciplinary team (MDT); Total intravenous anesthesia (TIVA); Electromagnetic navigation (DT-ENB); Positive end-expiratory pressure (PEEP); Rapid On-site Evaluation (ROSE); Virtual bronchoscopic navigation (VBN); Ultrathin bronchoscope (UTB); Image-guided bronchoscopy (IGB); Electromagnetic navigation (EMN) with integrated digital tomosynthesis (DT); Transthoracic puncture (TTP).
